# Treatment and Outcomes of COVID-19 Infection in Pregnant Women: Systematic Review of Cases Reported in Europe

**DOI:** 10.3390/jcm14113743

**Published:** 2025-05-27

**Authors:** Radica Živković Zarić, Milan Zarić, Simona Protrka, Veljko Andrić, Neda Arsenijević, Petar Čanović, Violeta Mladenović, Stefan Jakovljević, Miljan Adamović, Miona Glišić

**Affiliations:** 1 Department of Pharmacology and Toxicology, Faculty of Medical Sciences, University of Kragujevac, 34000 Kragujevac, Serbia; radica_zivkovic@yahoo.com; 2 University Clinical Center Kragujevac, 34000 Kragujevac, Serbia; velickovicneda@gmail.com (N.A.); vikicam2004@gmail.com (V.M.); stefan_jakov87@yahoo.com (S.J.); 3 Department of Biochemistry, Faculty of Medical Sciences, University of Kragujevac, 34000 Kragujevac, Serbia; petar.c89@gmail.com; 4 Department of Psychiatry, Faculty of Medical Sciences, University of Kragujevac, 34000 Kragujevac, Serbia; protrka.simona@gmail.com; 5 Department of Internal Medicine, Faculty of Medical Sciences, University of Kragujevac, 34000 Kragujevac, Serbia; andricraska@yahoo.com; 6 Department of Gynecology and Obstetrics, Faculty of Medical Sciences, University of Kragujevac, 34000 Kragujevac, Serbia; 7 Department of Surgery, Faculty of Medical Sciences, University of Kragujevac, 34000 Kragujevac, Serbia; 8 Department of Pharmacy, Faculty of Medical Sciences, University of Kragujevac, 34000 Kragujevac, Serbia; miljanadamovic84@gmail.com; 9 Pharmacy Institution “Zdravlje Lek”, 11000 Belgrade, Serbia; 10 Department of Dentistry, Faculty of Medical Sciences, University of Kragujevac, 34000 Kragujevac, Serbia; mionagrujovic@yahoo.com

**Keywords:** COVID-19 infection, pregnant women, treatment, outcome, newborns

## Abstract

**Background/Objectives:** The World Health Organization (WHO) declared a global pandemic of COVID-19 caused by SARS-CoV-2 in March 2020. May 2023 was the month that ended the global pandemic. Pregnant females with COVID-19 are less likely to be symptomatic than non-pregnant patients, with nearly three-quarters being without symptoms. According to previous studies, even if somebody develops symptoms, they are usually mild, most commonly coughing (41%), fever (40%), and dyspnea (21%). Our study aims to search the literature systematically, especially case series and case reports published in Europe, and to summarize results about the kind of COVID-19 therapy in pregnant women and about outcomes in mothers and newborns. **Methods**: Our systematic review was registered at the International Prospective Register of Systematic Reviews (PROSPERO) with CRD42024566838. We searched PubMed/MEDLINE, Google Scholar, Web of Science, Scopus, and Serbian Citation Index (SCIndeks). In this study, case reports or case series with open, complete text that included full clinical records of the individuals identified with infection in pregnancy, thought to be caused by COVID-19, were used. Case series or case reports were eliminated if they (1) did not contain a full clinical report for every patient, or (2) included an individual who suffered from another viral infection other than COVID-19, so the clinical course and the outcome could not be precisely defined. We evaluated reporting bias and attrition bias. **Results**: Our study included 32 published studies (eight case series and 24 case reports) that included 56 individual cases. The oldest patient was 50 years old, and the youngest was 19 years old. The most common symptom initially was dry cough (*n* = 23; 41%), followed by fever (*n* = 21; 37%) and dyspnea (*n* = 10; 17%). In three patients, a lower level of thrombocytes was reported, with the lowest level of 86 × 10^9^. The most frequently used drugs in pregnant women with COVID-19 infection were azithromycin, lopinavir/ritonavir, hydroxychloroquine, as well as corticosteroids. Twenty-two patients were on mechanical ventilation. After all this reported therapy, ten women died, as well as seven newborns. **Conclusions**: From our results, we can conclude that mechanical ventilation correlates with cesarean section performed more frequently, as well as with a higher mortality rate of neonates. There are no significant data related to transplacental transmission of the virus. Generally, mortality in our group of patients (mothers) was 17%, which is similar to the general population death from COVID-19 infection.

## 1. Introduction

The World Health Organization (WHO) declared a global pandemic of COVID-19 caused by SARS-CoV-2 in March 2020. May 2023 was the month that ended the global pandemic [1]. According to some studies, the novel coronavirus disease COVID-19 can be lethal for vulnerable inhabitants such as the elderly, patients with comorbidities, and pregnant women [2]. Gravid women have previously been at augmented risk of severe maternal and neonatal morbidity and mortality from related viral diseases. Pregnant females with COVID-19 are less likely to be symptomatic than non-pregnant patients, with nearly three-quarters being without symptoms [3]. Even if somebody develops symptoms, they are usually mild, most commonly including cough (41%), fever (40%), and dyspnea (21%) [4].

A healthy pregnancy needs changes in the pregnant woman’s immune system to recognize a genetically foreign fetus. These variations in the immune system and modifications in the cardiac, pulmonary, and other systems can increase vulnerability to illness and mortality with infection during gravidity [5].

Severe COVID-19 illness has been related to primary medical conditions. According to some studies, risk factors for severe disease include being overweight or obese, being 35 years old or older, having pre-existing comorbidity, and being Black or Asian [1,6]. Though the exact mechanisms by which pre-existing situations affect COVID-19 susceptibility and severity are unidentified, a durable suggestion now indicates the increased risks of COVID-19 infections for persons with medical conditions, such as lung and liver diseases, and diabetes [7]. According to Zhou et al., people with mental illnesses are at the highest risk of COVID-19 infection, whereas those with CVD (cardiovascular disease) are at a lower risk [2]. When a newborn infant tests positive for SARS-CoV-2, it can be difficult to determine whether the transmission was intrauterine (during pregnancy and before labour onset), intrapartum (during labour and delivery), or postpartum, both through interaction with the mother or others or over breastfeeding. Standards to assess whether intrauterine transmission has occurred have been developed and include documentation of maternal infection, confirmation of SARS-CoV-2 in the first 24 h of life, and signs of persistence of disease in the neonate. Even though intrauterine transmission of SARS-CoV-2 has been recognized, it seems to be sporadic [8,9,10]. The results are contradictory, but in general, according to some studies, the virus is transmitted from mother to child in 3 percent of cases [10,11,12].

Generally, considering the pandemic is over, we do not have information about treatment and outcomes according to published literature about pregnant women suffering from COVID-19 infection. We have not published systematic literature searches about this problem, especially after the pandemic ended.

Our study aims to systemically search the literature, especially case series and case reports published in Europe, and to summarize results about the kind of COVID-19 therapy in pregnant women as well as about outcomes in mothers and newborns.

## 2. Materials and Methods

Our systematic review was registered at the International Prospective Register of Systematic Reviews (PROSPERO) with CRD42024566838.

Studies considered for this systematic review had to accomplish the following criteria: (1) type of study—case series and case reports published in Europe; (2) characteristics of participants—patients any age, proven pregnancy, identification of the COVID-19 infection by PCR, medical course, management, and the outcome at the very least had to be available for all patients. The exclusion criteria were as follows: (1) studies that did not encompass a complete clinical report for each patient, (2) studies that included an individual who suffered from another viral infection other than COVID-19, so the clinical course and the outcome could not be precisely defined.

Three authors (R.Z.Z., M.Z., and P.C.) independently searched the following electronic databases with no language or date limit: PubMed/MEDLINE, Google Scholar, Web of Science, Scopus, and Serbian Citation Index (SCIndeks). We used the following search strategy: (“pregnancy” [MeSH Terms] AND (“COVID-19” [MeSH Terms] OR (“pregnancy” [All Fields] AND “COVID-19” [All Fields]) OR “pregnant women” [All Fields] OR (“pregnant women” [All Fields] AND “COVID-19” [All Fields]) OR (“pregnancy” [MeSH Terms] AND (“SARS-CoV-2” [MeSH Terms] OR (“pregnancy” [All Fields] AND “pregnancy” [All Fields]) OR “pregnant women” [All Fields] OR (“pregnant” [MeSH Terms] OR (“women” [All Fields] AND “SARS-CoV 2” [All Fields]).

### 2.1. Data Collection and Analysis

Primarily, the appropriateness of the included studies’ title and abstract was screened by five authors (R.Z.Z., M.Z., P.C., M.G., V.M.) autonomously. The complete text of the published study was protected and examined to determine whether the publication approved the study topic associated with the title and information provided in the abstract. If all authors agreed that the suitability standard had been met, studies were included in the systematic review. If there were divergences between separate authors, compromise was essential.

The following information was autonomously extracted for each case given by four authors (S.P., V.A., N.A., and S.J.): demographic information (age), study origin (country), total study duration, BMI of patients, week of gestation, information about previous deliveries, other conditions, the maximal level of CRP, the maximal level of procalcitonin (PCT), the maximal level of leucocytes, the maximal level of lymphocytes, level of thrombocytes, morphological diagnostics, presence of clinical signs of infection, presence of bacteria or fungi in the body fluids, antibiotic used, antivirotic used, other therapy used, outcome for mother, outcome for newborn. The primary outcomes were mother survival, newborn survival, and infection treatment.

### 2.2. Assessment of the Risk of Bias of Included Studies

Risk of bias was assessed by two investigators independently (M.Z. and P.C.), and the principal investigator (R.Z.Z.) made the last estimation. The following bases of bias were assessed: (1) reporting bias and (2) attrition bias [13].

First, reporting bias was assessed individually for each study, checking what proportion of target products was reported. Second, the geographical circulation of published studies was arranged and checked for consistency.

### 2.3. Measures of the Study Variables

The following outcomes were categorical: changes in laboratory parameters of an organ (or tissue) function suggestive of infection in that organ (or tissue), effects of antibiotic and antiviral management (cure rate and mortality), number of adverse events and category, antibiotics used, antiviral used, other therapy used, and morphological diagnostics which confirmed invasive infection (NMR, ultrasound, etc.). The following effects used in the study were continuous: total study duration, age of patients, total number of patients, maximal serum level of C-reactive protein during the disease, maximal level of procalcitonin in serum during the disease, maximal white blood cell count during the disease, as well as maximal level of thrombocytes and lymphocytes.

## 3. Results

**Figure 1:**
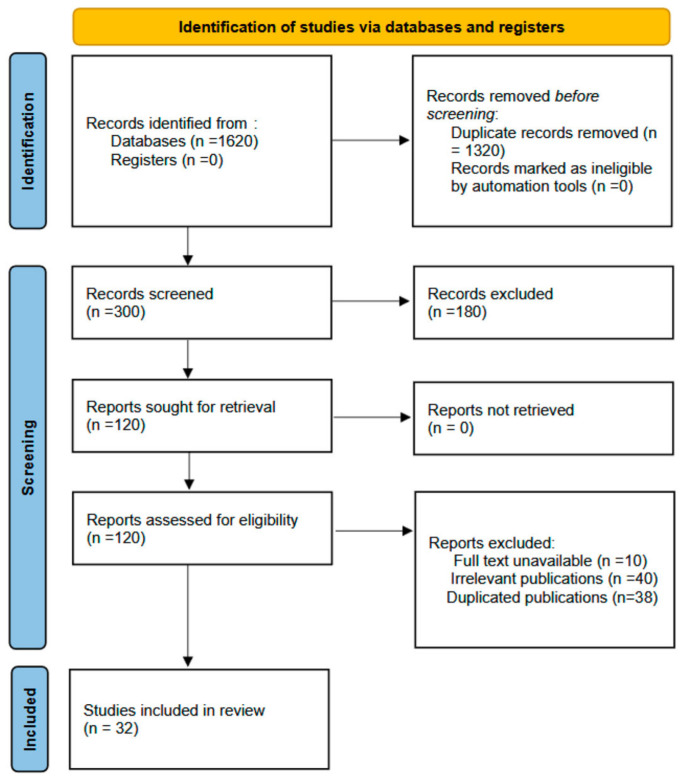
PRISMA flow diagram.

Our study included 32 published studies (eight case series and 24 case reports) (Figure 1) that included 56 individual cases. The oldest patient was 50 years old, and the youngest was 19 years old. The distribution of the years was as follows: patients in their twenties (*n* = 8; 14%), patients in their thirties (*n* = 38; 67%), patients in their forties (*n* = 8; 14%), as well as one patient who was 19 years old and one patient who was 50 years old. BMI ranged from 24 to 50. More than half of the cases were in the last trimester of pregnancy (*n* = 36; 62%). In most cases (*n* = 23; 41%), there were no data about previous deliveries; in 23%, there was no previous delivery. Most of the cases were described in Turkey (*n* = 12; 21%), followed by Serbia (*n* = 8; 14%), Italy (*n* = 6; 10%), and the United Kingdom (*n* = 6; 10%). Other cases were described in Portugal, The Netherlands, Austria, Poland, Croatia, France, Romania, Germany, Norway, and the Czech Republic. The characteristics of the included studies are shown in Table 1 and Table 2.

In most cases, patients had no other diseases or had diseases that were not reported (*n* = 33; 58%). The most common condition was anemia (*n* = 7; 12%), followed by hypertension, gestational diabetes, celiac disease, lupus erythematous, atrial septal defect, congenital adrenal hyperplasia, hypothyroidism, and DM type 2. In 27 cases, pneumonia was proven (CT and/ or RTG; lung ultrasound). The most common symptom initially was dry cough (*n* = 23; 41%), followed by fever (*n* = 21; 37%) and dyspnea (*n* = 10; 17%). Other symptoms were tachycardia and tachypnea, fatigue, and headache and in seven cases, there were no symptoms. In 26 patients, CRP was elevated, with a maximal value of 340 mg/L. In five patients, an elevated level of PCT was reported, with a maximal value of 90 ng/mL. In 16 patients, the level of leucocytes was higher, the maximal level of leucocytes was 29,000/mm^3^, and the highest level of lymphocytes was 16,000/mm^3^. In three patients, a lower level of thrombocytes was reported, with the lowest level of 86 × 10^9^. Only in eight cases were bacteria isolated from the patient’s sputum or blood (14%). There was *Haemophilus influenzae*, *Klebsiella pneumoniae*, MRSA (methicillin-resistant *Staphylococcus aureus*), *Burkholderia cepacia*, *Pseudomonas aeruginosa*, *Acinetobacter baumannii*, and *Serratia marcescens*.

**Figure 2:**
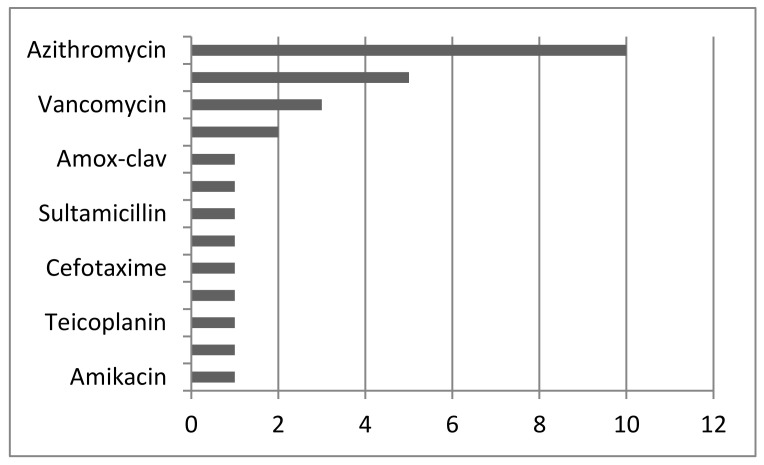
Antibiotics used in cases included in this study.

**Figure 3:**
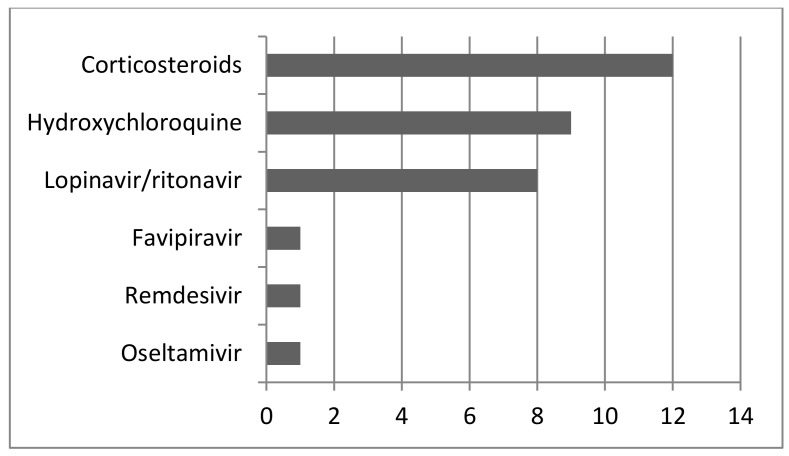
Other therapies used in cases included in this study.

Twelve patients had information about vaccination before infection (no vaccination) (21%). In other cases, there was not that information. In most cases (before delivery), azithromycin was used (*n* = 10; 17%), followed by piperacillin-tazobactam (*n* = 5; 8%). All used antibiotics are shown in Figure 2. The most commonly used antiviral drug was lopinavir-ritonavir (*n* = 8; 14%), followed by favipiravir (*n* = 2; 3%). Remdesivir and oseltamivir were also used. Hydroxychloroquine was used in nine patients (16%), Figure 3. In 14 patients (25%), corticosteroids were used. LWMH was used in 12 patients (21%). Twenty-two patients were on mechanical ventilation including ECMO (extracorporeal membrane oxygenation). Ten patients died (17%). Seven neonates died at delivery (12%). In 14 cases, we had no information for neonates (25%). Cesarean section was the most common type of delivery (*n* = 30; 53%). In eight cases, there was an ongoing pregnancy, so we do not know how it ended. One case was an abortion at 7 weeks of pregnancy, and in five cases, details about eventual delivery were not available.

## 4. Discussion

Coronavirus disease 2019 (COVID-19), produced by the severe acute respiratory syndrome coronavirus-2 (SARS-CoV-2), has posed an essential danger to public health. This virus distresses the respiratory area and, in most patients, typically leads to pneumonia and acute respiratory distress syndrome (ARDS) (15%). ARDS is one of the essential reasons for death in patients with COVID-19 and is mainly triggered by raised levels of pro-inflammatory cytokines, known as cytokine storm [46,47]. Since the commencement of the COVID-19 pandemic, more than 7 million people around the world have died of the illness, but the actual health burden of the pandemic is still far from being assessed [48,49].

According to our results, the most numerous were the patients in their thirties (67%). Of the patients, 62% were in the last trimester of pregnancy. The most common condition was anemia. In 27 cases, pneumonia was proven (CT and/or RTG; lung ultrasound) and the most common symptom initially was dry cough (41%). The maximal value of CRP was 340 mg/l. In most cases (before delivery), azithromycin was used (*n* = 10; 17%). The most commonly used antiviral drug was lopinavir-ritonavir (*n* = 8; 14%). From our group of patients included in this study, ten mothers died as well as seven neonates.

It is reasonable that most patients are in their thirties. First, today, the birth limit has shifted, so more women are deciding to have a child in their thirties, and then with age, the body’s resistance decreases, so there will be more cases of hospitalized patients than would have been the case in their twenties. Most of the reported cases were in the last trimester of pregnancy; this could be explained by the notion that the later the symptoms of pregnancy appear, the greater the chance of coronavirus infection causing symptoms. This correlates with published studies [50,51]. In 13 patients, BMI was above 30. From that group of patients, six died (in total, 10 patients died). We can conclude that obesity contributes to the poor outcome of pregnant women with coronavirus [52].

The most common condition in our patients was anemia, and in most cases, they were healthy before the COVID-19 infection. This may explain the lower mortality rate in our patient group. For example, diabetes mellitus as well as chronic hypertension could be related to poorer outcomes in such females [52]. In our group of patients, the most common symptom was cough (41%). This is relatively in correlation with other studies, e.g., Hammad et al. suggested dry cough in 51% of patients, and Dashraath et al. suggested 28% of patients had the same symptom [52,53]. Fever affected 31% of patients, and seven patients were asymptomatic. Pneumonia was diagnosed in 45% of the patients. In other studies, that number was about 76% [21]. In our study in 22 patients from this group, mechanical ventilation was included, and in the Dashraath et al. study [53], only two patients were on mechanical ventilation. We could explain this, maybe, with the fact that the patients from our group saw the doctor later, and the pneumonia was already advanced. From the group of patients with mechanical ventilation, 50% of patients died, which is the average for patients on mechanical ventilation. According to Sison et al., mortality rates vary across different care settings ranging broadly from 13.7% to 77.8% in ICUs, 7.8 to 51.0% in non-ICUs, and 12.0 to 91.8% in home/NH settings [54].

Cesarean section was the most common type of delivery in 53% of patients. This result is similar to other studies, e.g., Xu et al. [55], in their systematic review, showed a higher rate of cesarean section in the infected group compared to the control group, and in six studies, reported the probability of cesarean section only in the COVID-19 infected group, ranging from 15.5 to 38.5%. They also showed significant modifications in the style of delivery between infected and non-infected females, with COVID-19-positive females being more likely to experience cesarean section and deliver preterm infants [55]. Globally, cesarean section rates have increased annually, from 12.1% in 2000 to 21.1% in 2015 [56], and this rate is significantly higher than the World Health Organization’s (WHO’s) suggested suitable cesarean section rate of 10–15%. In our results, there was a higher percentage of cesarean delivery, as expected, so the mortality in newborns is 7%. Only in eight cases were bacteria isolated from the patient’s sputum or blood (14%). The presence of bacteria in the body may be associated with a worse prognosis for patient survival [57]. Only Beric et al., Craina et al., and Mihajlovic et al. [18,20,30] reported information about the vaccination status of those patients (21%). In other cases, there was no such information, probably because most of the manuscripts are from 2020 and 2021, and the vaccine began to be used more widely in late 2020 and early 2021. So, vaccination was not yet as widespread when the cases were published [58].

Hydroxychloroquine (HCQ), a 4-aminoquinoline derivative with antimalarial and immune-modulatory properties, has been in use for years for the treatment of malaria and immune disorders like systemic lupus erythematosus [59]. In our group of patients, hydroxychloroquine was used in nine patients (16%). Generally, according to some authors, there is a slight increase in the risk of malformations associated with first-trimester hydroxychloroquine use. In the third trimester, it is safe for pregnancy [60].

Lopinavir/ritonavir is the most frequently used antiviral drug (14%). It is an antiviral combination that belongs to the group of protease inhibitors (PIs).

It has in vitro and in vivo efficacy against SARS-CoV-1 and MERS infections, so they were considered a management choice for SARS-CoV-2 infection. There is extensive experience of its use in pregnant women with HIV infection. This pharmacological combination has a slight transplacental passage. The optional dosage is twice a day, and the dose in the third trimester is increased if needed [61]. Twelve percent of neonates died at delivery. In 14 cases, we do not have information about the neonates (25%). From Table 2, we can see that in cases with mechanical ventilation, cesarean sections were performed in more than half of the patients (*n* = 14; 63%). All neonates who died were in the group of patients on mechanical ventilation. From the same group of patients, of the neonates who survived, six had a lower body weight (less than 2.5 kg). Those neonates also had worse Apgar scores. In our studies, there was no specific date about the transplacental transmission of infection. An Italian study analyzed the impact of placental impairment on perinatal outcomes [62]. It concluded that there is an absence of specific pathological findings for SARS-CoV-2 infections. Nevertheless, a high number of placentas showed signs of inflammation, which may be related to a storm of cytokines induced by the virus, without important perinatal concerns. A study from Greece showed slightly different results [63].

Mortality in our group of patients (mothers) was 17%, which is similar to the general population death rate [64]. After all, it is not possible to explicitly state that this is because of the COVID-19 infection, because autopsies were generally not performed, or at least not stated, but given that the papers were published with titles related to COVID-19 (as well as conclusions of the manuscripts), it is assumed that in these cases, the mortality was related to this viral infection.

An Iranian study found a statistically significant difference in maternal death rates between the COVID-19 group and the control group [65]. Some studies showed no significant difference in maternal mortality rates between the COVID-19 and control groups [62]. A retrospective observational study from Dubai [66] divided COVID-19 patients into mild/moderate and severe clusters, and the conclusion was that the death rate was higher among gravid women with severe COVID-19.

## 5. Conclusions

We can conclude that the most frequently used drugs in pregnant women with COVID-19 infection were azithromycin, lopinavir/ritonavir, hydroxychloroquine, as well as corticosteroids. After all this reported therapy, ten women died, as well as seven newborns. From our results, mechanical ventilation correlates with cesarean section performed more frequently, as well as with a higher mortality rate of neonates. Survived newborns generally had a solid Apgar score at the first and fifth minutes and a good prognosis. There are no significant results on the impact of vaccination on the outcome of infection. Also, there are no significant data related to transplacental transmission of the virus. Generally, mortality in our group of patients (mothers) was 17%, which is similar to the general population death rate.

A limitation of this study may be that the cases are only from Europe and that these are conclusions from published cases when, in reality, there were many more. In the future, more systematic review papers on this topic should be published, which, depending on the author’s interests, would also include other types of studies to see if there are any differences in the results.

## Figures and Tables

**Table 1:** Demographic characteristics of study sample.

No of Case	Publication ID	Study Design	Attrition/Reporting Bias	Age and BMI	Length of Hospitalization (Days)	Week of Pregnancy When COVID-19 Was Diagnosed	Morphological Diagnosis of Pneumonia
1	Agbontaen et al., 2021 [14]	Case report	Low/Low	42/27.6	54	24	CT
2	Ambroz et al., 2022 [15]	Case report	High/High	32/NA	NA	32	CT
3	Anness et al., 2020 [16]	Case report	Low/Low	35/NA	160	28	RTG
4	Baglı et al., 2021 [17]	Case series	Low/Low	34/26	NA	34	CT
5	Baglı et al., 2021 [17]	Case series	Low/Low	37/28	NA	36	CT
6	Baglı et al., 2021 [17]	Case series	Low/Low	33/33	NA	33	CT
7	Baglı et al., 2021 [17]	Case series	Low/Low	39/30	NA	35	CT
8	Beric et al., 2023 [18]	Case report	High/Low	35/NA	47	28	CT and RTG
9	Clemenza et al., 2022 [19]	Case series	Low/Low	27/NA	120	18	LUS
10	Clemenza et al., 2022 [19]	Case series	Low/Low	38/NA	60	28	LUS
11	Clemenza et al., 2022 [19]	Case series	Low/Low	43/38	90	38	LUS
12	Craina et al., 2022 [20]	Case series	Low/Low	35/26	NA	38	NA
13	Craina et al., 2022 [20]	Case series	Low/Low	21/26	NA	39	NA
14	Craina et al., 2022 [20]	Case series	Low/Low	35/26	NA	39	NA
15	De Nardo et al., 2021 [21]	Case report	High/High	36/NA	30	40	NA
16	Esposito et al., 2023 [22]	Case report	Low/Low	34/NA	60	19	CT
17	Federici et al., 2020 [23]	Case report	Low/Low	33/NA	20	23	RTG
18	Figuerido et al., 2020 [24]	Case report	Low/Low	35/39	20	39	NA
19	Fiore et al., 2021 [25]	Case report	High/Low	31/31	NA	31	RTG and CT
20	Giavioli et al., 2021 [26]	Case report	High/Low	39/35.2	35	36	CT
21	Lyra et al., 2020 [27]	Case report	High/Low	35/NA	3	39	None
22	Maier et al., 2020 [28]	Case report	Low/Low	38	NA	38	RTG and CT
23	Manau et al., 2020 [29]	Case series	High/High	50/25	17	22	RTG
24	Manau et al., 2020 [29]	Case series	High/High	30/32	22	20	RTG
25	Mihajlovic et al., 2022 [30]	Case series	High/Low	42/25.6	NA	29	NA
24	Mihajlovic et al., 2022 [30]	Case series	High/Low	29/24	NA	27	NA
27	Mihajlovic et al., 2022 [30]	Case series	High/Low	32/30	NA	33.6	NA
28	Mihajlovic et al., 2022 [30]	Case series	High/Low	38/32	NA	27	NA
29	Mihajlovic et al., 2022 [30]	Case series	High/Low	33/27	NA	33	NA
30	Mihajlovic et al., 2022 [30]	Case series	High/Low	26/46	NA	29.33	NA
31	Mihajlovic et al., 2022 [30]	Case series	High/Low	30/32	NA	29.4	NA
32	Mihajlovic et al., 2022 [30]	Case series	High/Low	30/32	NA	14	NA
33	Munoz et al., 2023 [31]	Case report	Low/High	40/NA	NA	23	RTG
34	Palailoglu et al., 2021 [32]	Case report	Low/Low	42/NA	NA	37	CT
35	Palmrich et al., 2021 [33]	Case report	Low/Low	36/50	13	27	RTG
36	Pikovsky et al., 2021 [34]	Case series	Low/Low	34/25	12	35	CT
37	Pikovsky et al., 2021 [34]	Case series	Low/Low	34/28	5	36	CT
38	Ravn et al., 2021 [35]	Case report	High/High	30/NA	NA	33	CT
39	Reindorf et al., 2021 [36]	Case report	Low/Low	35/NA	5	27	RTG
40	Rodrigues et al., 2020 [37]	Case report	High/High	35/NA	3	40	CT
41	Rosner Tenerowich et al., 2021 [38]	Case report	Low/Low	38/NA	60	27	NA
42	Sileo et al., 2021 [39]	Case report	High/Low	30/NA	NA	38	None
43	Smeele et al., 2021 [40]	Case series	High/Low	31/NA	2	39	None
44	Smeele et al., 2021 [40]	Case series	High/Low	39/NA	70	19	None
45	Sokolov et al., 2022 [41]	Case report	High/Low	37/NA	NA	34	RTG
46	Stout et al., 2020 [42]	Case report	Low/Low	28/24	19	36	RTG and CT
47	van Amesfort et al., 2021 [43]	Case report	Low/Low	21/NA	15	37	CT
48	Vibert et al., 2020 [44]	Case report	High/High	21/NA	NA	23	CT
49	Yassa et al., 2021 [45]	Case series	Low/High	32/NA	NA	39	LUS
50	Yassa et al., 2021 [45]	Case series	Low/High	32/NA	NA	27	LUS
51	Yassa et al., 2021 [45]	Case series	Low/High	33/NA	NA	20	LUS and CT
52	Yassa et al., 2021 [45]	Case series	Low/High	19/NA	NA	9	LUS
53	Yassa et al., 2021 [45]	Case series	Low/High	41/NA	NA	17	LUS and CT
54	Yassa et al., 2021 [45]	Case series	Low/High	40/NA	NA	7	LUS and CT
55	Yassa et al., 2021 [45]	Case series	Low/High	23/NA	NA	10	LUS and RTG
56	Yassa et al., 2021 [45]	Case series	Low/High	40/NA	NA	38	LUS and CT

Abbreviations: NA—not available; LUS—lung ultrasound; CT—computerized tomography; RTG—radiography; BMI—body mass index.

**Table 2:** Outcome of the reported cases.

No of Case	Publication ID	Antibiotic Treatment of the Patient BD	Antivirotic Treatment of the Patient BD	Respiratory Support; Method of Delivery	Outcome of the Mother and the Children (Weight and Apgar Score at 1 and 5 Min)
1	Agbontaen et al., 2021 [14]	Piperacilin tazobactam, clindamycin; meropenem and amikacin	NA	No; CS	Survived; survived 1164 g
2	Ambroz et al., 2022 [15]	None	None	MV; CS	Survived; death
3	Anness et al., 2020 [16]	NA	NA	No; ND	Survived; survived 3200 g
4	Baglı et al., 2021 [17]	Vancomycin	Lopinavir-ritonavir	No; CS	Died; survived 2500; 5, 8
5	Baglı et al., 2021 [17]	None	None	MV; CS	Died; survived 3000; 6, 8
6	Baglı et al., 2021 [17]	Klaritromicine; Carbapenems	Lopinavir-ritonavir	MV; CS	Died; survived 2000; 7, 8
7	Baglı et al., 2021 [17]	Piperacillin tazobactame and teicoplanin	Lopinavinir-ritonavir	MV; CS	Died; survived 2250; 8, 9
8	Beric et al., 2023 [18]	NA	NA	MV, ECMO; CS	Survived; NA
9	Clemenza et al., 2022 [19]	Piperacillin-tazobactam and azithromycin; vancomycin	NA	ECMO; CS	Survived; survived 9 and 92,670 g
10	Clemenza et al., 2022 [19]	Oxacilin; vancomycin; piperacillin tazobactam	NA	ECMO; ND	Survived; survived 1 and 7; weighed 1880 g
11	Clemenza et al., 2022 [19]	NA	NA	MV, ECMO; CS	Survived; survived 9 and 10; baby weighed 3080 g
12	Craina et al., 2022 [20]	None	None	No; ND	Survived; survived 2990 g, 8, 10
13	Craina et al., 2022 [20]	None	None	No; CS	Survived; survived: 9, 10
14	Craina et al., 2022 [20]	None	None	No; CS	Survived; Survived; 9, 10
15	De Nardo et al., 2021 [21]	None	NA	No; ND	Survived; 9 and 10 and 3290 g
16	Esposito et al., 2023 [22]	NA	NA	MV; ND	Survived; Death
17	Federici et al., 2020 [23]	cefotaxime and spiramycin	NA	MV; ND	Survived; Apgar score NA, 3300 g
18	Figuerido et al., 2020 [24]	NA	NA	No; ND	Survived; 5-min Apgar Score of 10, 2870 g
19	Fiore et al., 2021 [25]	NA	NA	MV, ECMO; CS	Survived; survived 1680 g.
20	Giavioli et al., 2021 [26]	Azythromicin	NA	MV; CS	Survived; NA
21	Lyra et al., 2020 [27]	NA	NA	No; CS	Survived; survived Apgar score 8, 3110 g
22	Maier et al., 2020 [28]	Sultamicillin	None	MV; ND	Survived; NA
23	Manau et al., 2020 [29]	Azithromycin	Lopinavir-ritonavir	MV; OP	Survived; NA
24	Manau et al., 2020 [29]	None	None	No; OP	Survived; NA
25	Mihajlovic et al., 2022 [30]	NA	NA	MV; NA	Death; death
24	Mihajlovic et al., 2022 [30]	NA	NA	MV; CS	Survived; Survived
27	Mihajlovic et al., 2022 [30]	NA	NA	MV; NA	Death; Death
28	Mihajlovic et al., 2022 [30]	NA	NA	MV; NA	Death; Death
29	Mihajlovic et al., 2022 [30]	NA	NA	MV; CS	Survived; Survived
30	Mihajlovic et al., 2022 [30]	NA	NA	MV; NA	Death; Death
31	Mihajlovic et al., 2022 [30]	NA	NA	MV; CS	Survived; Survived
32	Mihajlovic et al., 2022 [30]	NA	NA	MV; NA	Death; Death
33	Munoz et al., 2023 [31]	NA	None	O2; ND	Survived; survived 2750 g, the Apgar score was 10,
34	Palailoglu et al., 2021 [32]	None	None	No; CS	Survived; 3580 g, 8, 10
35	Palmrich et al., 2021 [33]	None	Remdesivir	MV; CS	Survived; survived Apgar score of 3/7/7 at 1, 5, and 10 min, 1445 g
36	Pikovsky et al., 2021 [34]	NA	NA	O2; CS	Survived; Apgars of 8/9/10 at 1, 5, and 10 min, 3100 g
37	Pikovsky et al., 2021 [34]	NA	NA	No; CS	Survived; survived Apgar score NA, 3200 g
38	Ravn et al., 2021 [35]	Cefuroxime	None	No; CS	Survived; NA
39	Reindorf et al., 2021 [36]	Co-amoxicllav	NA	O2; ND	Survived; NA
40	Rodrigues et al., 2020 [37]	NA	NA	No; CS	Survived; NA
41	Rosner Tenerowich et al., 2021 [38]	Piperacillin tazobactam and azithromycin	Remdesivir	ECMO; CS	Survived; Apgar score of 7, 1440 g
42	Sileo et al., 2021 [39]	None	None	No; CS	Survived; survived 8, 8: 2680 g
43	Smeele et al., 2021 [40]	NA	NA	No; ND	Survived; survived Apgar score 9, 2880 g
44	Smeele et al., 2021 [40]	NA	NA	No; CS	Survived; survived Apgar score 10, 4205 g
45	Sokolov et al., 2022 [41]	None	None	O2; CS	Survived; survived the first baby 1400 g, 7 and 8; the second baby 1600 g, 8; the third baby was 1820 g, 7 and 8; the fourth baby was 1520 g, 6 and 8
46	Stout et al., 2020 [42]	NA	NA	ECMO; CS	Death; survived, Apgar score 8
47	van Amesfort et al., 2021 [43]	NA	NA	No; CS	Survived; survived 2930 g, 7 and 8
48	Vibert et al., 2020 [44]	NA	NA	O2; OP	Survived; NA
49	Yassa et al., 2021 [45]	NA	None	No; CS	Survived; survived 3070-g, 8 and 9.
50	Yassa et al., 2021 [45]	Azithromycin	Oseltamivir	No; OP	Survived; NA
51	Yassa et al., 2021 [45]	Azithromycin; meropenem	Lopinavir/ritonavir; favipiravir	No; OP	Survived; NA
52	Yassa et al., 2021 [45]	None	Ritonavir/lopinavir	No; OP	Survived; NA
53	Yassa et al., 2021 [45]	Azithromycin	Ritonavir/lopinavir	No; OP	Survived; NA
54	Yassa et al., 2021 [45]	Azithromycin	None	No; Abortion	Survived
55	Yassa et al., 2021 [45]	Azithromycin	Ritonavir/lopinavir	No; OP	Survived; NA
56	Yassa et al., 2021 [45]	None	None	No; CS	Survived; NA

Abbreviations: CS—cesarean section; ND—natural (vaginal) delivery; OP—ongoing pregnancy; BD—before delivery; MV—mechanical ventilation; ECMO—extracorporeal membrane oxygenation; NA—not available.
